# Association of sleep duration and insomnia with metabolic syndrome and its components in the Women’s Health Initiative

**DOI:** 10.1186/s12902-022-01138-9

**Published:** 2022-09-14

**Authors:** Rita Peila, Xiaonan Xue, Elizabeth M. Cespedes Feliciano, Matthew Allison, Susan Sturgeon, Oleg Zaslavsky, Katie L. Stone, Heather M. Ochs-Balcom, Yasmin Mossavar-Rahmani, Tracy E. Crane, Monica Aggarwal, Sylvia Wassertheil-Smoller, Thomas E. Rohan

**Affiliations:** 1 Department of Epidemiology and Population Health, Albert Einstein College of Medicine, 1300 Morris Park Avenue Belfer, Rm1301A, Bronx, NY 10461 USA; 2 Division of Research, Kaiser Permanente Division of Research, Oakland, CA USA; 3 Division of Preventive Medicine, University of California, San Diego, CA USA; 4 Institute of Applied Life Sciences, University of Massachusetts, Amherst, MA USA; 5 Department of Biobehavioral Nursing and Health Informatics, University of Washington, Seattle, WA USA; 6 California Pacific Medical Center Research Institute, San Francisco, CA USA; 7 Department of Epidemiology and Environmental Health, University of Buffalo, Bufallo, NY USA; 8 Behavioral Measurement and Interventions Cancer Prevention and Control Program, University of Arizona, Tucson, AZ USA; 9 Division of Cardiology, University of Florida, Gainesville, FL USA

**Keywords:** Postmenopausal women, Sleep duration, Insomnia, Metabolic syndrome, Longitudinal study

## Abstract

**Background:**

Epidemiological evidence suggests that inadequate sleep duration and insomnia may be associated with increased risk of metabolic syndrome (MetS). However, longitudinal data with repeated measures of sleep duration and insomnia and of MetS are limited. We examined the association of sleep duration and insomnia with MetS and its components using longitudinal data from the Women’s Health Initiative (WHI).

**Methods:**

The study included postmenopausal women (ages 50–79 years) diabetes-free at enrollment in the WHI, with baseline data on sleep duration (*n* = 5,159), insomnia (*n* = 5,063), MetS, and its components. Repeated measures of self-reported sleep duration and insomnia were available from years 1 or 3 of follow-up and of the MetS components from years 3, 6 and 9. Associations were assessed using logistic regression and generalized estimating equations models, and odds ratios and 95% confidence intervals (CI) adjusted for major risk factors were calculated**.**

**Results:**

In cross-sectional analysis, baseline sleep duration ≥ 9 h was positively associated with MetS (OR = 1.51; 95%CI 1.12–2.04), while sleep duration of 8- < 9 h was associated with waist circumference > 88 cm and triglycerides ≥ 150 mg/dL (OR = 1.18; 95%CI 1.01–1.40 and OR = 1.23; 95%CI 1.05–1.46, respectively). Insomnia had a borderline positive association with MetS (OR = 1.14; 95%CI 0.99–1.31), and significant positive associations with waist circumference > 88 cm and glucose ≥ 100 mg/dL (OR = 1.18; 95%CI 1.03–1.34 and OR = 1.17; 95%CI 1.02–1.35, respectively). In the longitudinal analysis, change from restful sleep to insomnia over time was associated with increased odds of developing MetS (OR = 1.40; 95%CI 1.01–1.94), and of a triglyceride level ≥ 150 mg/dL (OR = 1.48; 95%CI 1.08–2.03).

**Conclusions:**

Among postmenopausal women in the WHI, sleep duration and insomnia were associated with current and future risk of MetS and some of its components.

**Supplementary Information:**

The online version contains supplementary material available at 10.1186/s12902-022-01138-9.

## Background

Poor sleep quality with difficulty initiating sleep, maintaining sleep, and/or not obtaining restful sleep, affects multiple body systems and can impair physical and mental health [1]. Relatively short and long sleep duration have been identified as predictors of outcomes such as cardiovascular disease, stroke [2], cancer [3], reduced pain tolerance [4], impaired cognitive function [5], and mortality [2, 6]. The American Academy of Sleep Medicine and Sleep Research Society recommends that adults have at least seven hours of sleep per night [7].

Metabolic syndrome (MetS) is defined by the presence of metabolic abnormalities such as hyperglycemia, abdominal obesity, hypertension and dyslipidemia (low HDL cholesterol and/or high triglycerides (TG)). In recent decades, the prevalence of MetS in the United States adult population has increased steadily, reaching approximately 35% [8].

Epidemiological studies suggest that both sleep duration and insomnia are associated with MetS. The results of a meta-analysis of several cross-sectional studies showed increased risk of MetS in those with shorter sleep duration (approximately < 6.5 h) compared to individuals with 7- < 8 h of sleep [9], with the majority of these studies also reporting an association between longer sleep duration (> 9 h) and an increased risk of MetS [10–13]. Furthermore, a recent meta-analysis indicated that poor sleep quality is associated with MetS [14].

Few longitudinal studies have reported on the relationship between sleep duration, insomnia and MetS. One study analyzed the association between changes over time in sleep duration and MetS [15], while none has examined the association of changes in insomnia with MetS. In the present study, we conducted both cross-sectional and longitudinal analyses of the relationship between sleep quantity and insomnia and risk of MetS and its components in a sub-group of women within the Women’s Health Initiative (WHI).

## Methods

### Study population

The WHI is a population-based, multi-race/ethnic, multicenter prospective study of postmenopausal women, aged 50 to 79 years at enrollment (1993–1998) [16], and includes an observational component (OS, *n* = 93,676), and a clinical trial component (CT, *n* = 68,132) which consisted of four trials: dietary modification, calcium/vitamin-D supplementation, and hormone therapy (two trials). Details on the study design, eligibility, and protocols have been reported [17]. The study was approved by the institutional Ethical Review Board at each study center and by the Coordinating Center at the Fred Hutchinson Cancer Research Center, and was conducted in accordance with the Declaration of Helsinki. Participants provided written informed consent prior to being enrolled [18, 19].

The present study included 5,606 women (a 6% random sample of the CT, *n* = 4,544, and a 1% sub-sample of the OS, *n* = 1,062) who were selected for different WHI sub-studies designed to evaluate cardiovascular biomarkers [20]. The 6% sample of the CT was stratified by age, clinical center, and hysterectomy status, with oversampling of minority groups to increase the percentage of minorities compared to the CT cohort; the 1% sample of the OS was selected randomly from that cohort.

### Exposure assessment

Baseline demographic, lifestyle and medical information was collected using self-administrated questionnaires.

Sleep duration was self-reported as hours/night (≤ 6, 6- < 7, 7- < 8, 8- < 9, 9- < 10, ≥ 10) in response to the question, “About how many hours of sleep did you get on a typical night during the past four weeks?” Sleep quality was assessed using the WHI Insomnia Rating Score (WHIIRS), which was developed and validated in the WHI [21]. The WHIIRS is a 5-item instrument to measure: 1) trouble falling asleep, 2) waking up several times during the night, 3) waking up earlier than planned, and 4) trouble getting back to sleep and 5) overall rating of how restful sleep was during the past 4 weeks. Each item was measured on a 0–4 scale, with higher scores representing more frequent or severe symptoms. A final score (0–20), was calculated by summing the scores for the 5 items. Insomnia was defined as a score ≥ 9 [21], a cut-point that has been used in the WHI cohort to examine the association between sleep quality and cardiovascular health [22]. Sleep duration and insomnia measures were obtained at baseline and at years 3 for the whole sample (OS and CT), and additionally at year 1 for the CT.

### Additional measurements

Variables collected at baseline also included age, race/ethnicity, education (< high school, high school graduate, some college, and college graduate), marital status (married or marriage-like relationship, widowed, divorced or separated, or single), family income, chronic disease (cancer, diabetes, cardiovascular), history of cigarette smoking (never, former and current), pack-years of cigarette smoking (years smoked multiplied by the average number of cigarettes/day), alcohol consumption (no alcohol, < 1 serving/week, 1–7 serving/week, daily), coffee and tea consumption (cups/day), and physical activity (calculated by multiplying the hours/week of each reported leisure-time physical activity by the metabolic equivalent of that activity, and summing across activities). At the baseline visit, participants were asked about the frequency of use of “any kind of medication or alcohol at bedtime to help you sleep” in the past 4 weeks (no, < 1x/week, 1-2x/week, 3-4x/week, ≥ 5x/week). Diabetes was defined as having a physician diagnosis or taking diabetes medication. Depression was measured using the Center for Epidemiological Studies of Depression (CES-D) scale; a score ≥ 5 on the CES-D scale short version was used to define depressive symptoms [23]. At baseline and follow-up exams, weight was measured with a balance beam scale to the nearest 0.1 kg, and height was measured with a stadiometer to the nearest 0.1 cm. Weight and height were obtained by trained and certified clinical center personnel. Body mass index (BMI) was calculated as weight (kg)/square of height (m^2^).

### Metabolic syndrome

We used the MetS definition for women proposed by the National Cholesterol Education Program’s Adult Treatment Panel-III report (NCEP/ATP-III) [24], defined as having ≥ 3 of the following five criteria: WC > 88 cm, fasting glucose (FG) ≥ 100 mg/dL or diabetes medication, hypertension defined as systolic BP ≥ 130 or diastolic BP ≥ 85 mmHg or use of anti-hypertension medication, high density cholesterol (HDL) < 50 mg/dL or cholesterol lowering medication, and TG ≥ 150 mg/dL.

Fasting blood samples were collected at baseline in the whole cohort, plus at years 1, 3, and 6 post-randomization fin the CT sample, and at year 3in the OS sample. The blood specimens were processed and serum and plasma were stored at -70 °C within 2 h of collection. Analyte measurements were performed at Medical Research Laboratories Inc. (Highland Heights, KY). Serum was used to measure fasting glucose, using the hexokinase method on the Hitachi 747,cholesterol using a cholesterol oxidase method (Roche Diagnostics) on the Roche Modular P Chemistry analyzer, and TG, using Triglyceride GB reagent (Roche Diagnostics) [16, 25]. Waist circumference was measured to the nearest 0.1 cm at the narrowest part of the torso. Blood pressure was measured in the upper right arm using an appropriate cuff size and a mercury sphygmomanometer after the participant was seated and rested for 5 min; the average of two BP measurements (≥ 30 min apart) was then used. Waist circumference (WC) and blood pressure were measured at baseline and at years 1, 3, 6, and 9.

### Analytic sample

Since diabetes is associated with sleep disturbance and with development of metabolic dysregulation [26], we excluded from the 5,606 participants with biomarkers data all women who reported a history of diabetes at baseline (*n* = 411). Furthermore, we excluded 36 women with missing baseline information on both sleep duration and insomnia and additional 96 women with missing data on insomnia only. The final sample included 5,159 women with data on sleep duration, of these, 5,063 had information on insomnia (Supplementary Table 1).

### Statistical analysis

Baseline characteristics were compared by MetS status (no/yes) using Wilcoxon rank-sum tests for continuous variables and chi-square tests for categorical variables.

Cross-sectional associations of baseline sleep duration and insomnia with MetS and its components were evaluated, separately or with both exposure variables in the same model, using logistic regression to estimate odds ratios (OR) and 95% confidence intervals (CI). Sleep duration was categorized as follows: < 6, 6- < 7, 7- < 8, 8- < 9, and ≥ 9 h, and insomnia as a WHIIRS score of < 9 or ≥ 9. For the main analysis of sleep duration, we used 7- < 8 h of sleep as the referent category as many studies have shown that postmenopausal women in this group has the lowest morbidity and mortality [6, 27–29]. The group with 7- < 8 h/no-insomnia was selected as referent to test the joint association of sleep duration and insomnia with the outcomes. In addition, we performed a sensitivity analysis using sleep duration of 7- < 9 h as the referent category.

Generalized estimating equation (GEE) models with an exchangeable working correlation were used to evaluate the longitudinal associations of the exposures with risk of MetS. With GEE analysis, the association between two longitudinally-measured variables can be studied using all longitudinal data simultaneously, adjusting for possible within-person correlations between the repeated measurements on each participant, with robust estimation of the variances. Age at each exam was used as a time varying variable. Women who developed diabetes before the occurrence of the outcome of interest (*n* = 394) were excluded from these analyses. In this analysis, the mean hours of sleep between baseline and the second visit (year 1 for the CT group, and year 3 for the OS group) was used as the estimate of sleep duration over time. Participants with MetS at baseline or at the second visit were excluded from the longitudinal analysis. Women who died during follow-up contributed up to the last exam they attended. All the analyses for this study were performed in 2020. Changes in sleep duration were calculated by subtracting the value reported at baseline from the value at the second visit. For the majority of the subjects (92%), sleep duration was similar at the two visits (within 2 h of each other) and their mean value was categorized as in the cross-sectional analysis using 7- < 8 h of sleep as referent category in the analysis. Women with sleep duration changes ≥ 2 h between these two visits, corresponding to at least 2 standard deviations of the distribution of the observed change, were included in two distinct categories (≥ 2 h increase and ≥ 2 h decrease) [15]. As with the cross-sectional analysis, a sensitivity analysis was performed using 7- < 9 h of sleep as referent category. In addition, persistence/change in insomnia over time was based on its status at baseline and the first follow-up visit. Four mutually exclusive groups were created and defined as: “stable, restful sleep” (referent category), “persistent insomnia”, and changed from “restful sleep to insomnia”, or “insomnia to restful sleep”. All models were adjusted for age, race, study arm, education, smoking status, pack-years of cigarette smoking, alcohol intake, hormone use, physical activity, depression, age at menopause and marital status. Because BMI is correlated with WC (Pearson’s *r* = 0.82, *p*-value < 0.001) and MetS, it was not included in the main analysis, but added in sensitivity analyses.

## Results

Table 1 shows the baseline characteristics by presence/absence of MetS. Higher BMI, depression, relatively low alcohol consumption, lower levels of physical activity, longer sleep hours, insomnia, and more frequent use of sleeping aids were more prevalent in women with MetS than in those without MetS.

**Table 1:** Baseline characteristics by metabolic syndrome status

	Metabolic Syndrome ^a^		
	Yes	No	*p-value*
n, %	1,656 (30.79)	3,497 (69.21)	
Age, years	63 (11)	62 (12)	0.006
Sleep duration (hours), n (%) < 6	202 (12.20)	417 (11.92)	
6- < 7	501 (30.25)	1,040 (29.74)	
7- < 8	543 (32.79)	1,236 (35.24)	
8- < 9	323 (19.50)	679 (19.42)	
≥ 9	87 (5.25)	125 (3.57)	0.040
Insomnia (IRS ≥ 9), n (%) ^b^	517 (31.78)	995 (29.01)	0.045
Waist circumference > 88 cm	1,329 (80.40)	898 (25.79)	< 0.001
Hypertension, n (%)	1,358 (82.00)	1,482 (42.38)	< 0.001
Fast glucose ≥ 100 mg/dL	984 (59.82)	402 (11.88)	< 0.001
HDL < 50 mg/dL	1,053 (63.86)	396 (11.73)	< 0.001
Triglycerides ≥ 150 mg/dL	1,181 (71.40)	697 (20.60)	< 0.001
Observational Study, n (%)	227 (13.71)	756 (21.62)	< 0.001
Dietary modification trial, n (%)	888 (53.62)	1,706 (48.78)	0.001
Calcium and Vitamin D suppl. trial, n (%)	728 (43.96)	1,474 (42.15)	0.220
HRT study arm, n (%)	910 (54.95)	1,546 (44.21)	< 0.001
Use of sleep aids no	1,274 (76.93)	2,778(79.44)	
< 1t/wk	127 (7.67)	312 (8.92)	
1-2t/wk	84 (5.07)	161 (4.60)	
3-4t/wk	46 (2.78)	84 (2.40)	
≥ 5t/wk	120 (7.25)	157 (4.49)	0.001
Body mass index, kg/m^2^	31.59 (7.27)	26.20 (6.27)	< 0.001
Education< college	168 (10.24)	252 (7.25)	
some college	1,033 (62.99)	1,891 (54.39)	
college graduate	133 (8.11)	372 (10.70)	
post-college	306 (18.66)	962 (27.67)	< 0.001
Ethnicity, n (%) White	851 (51.39)	1,918 (54.85)	
Black	392 (23.67)	752 (21.50)	
American Indian/Alaskan Native	52 (3.1)	66 (1.9)	
Asian or Pacific Islander	99 (6.0)	289 (8.3)	
Hispanic/Latino	237 (14.3)	409 (11.7)	
Others	25 (1.5)	63 (1.8)	< 0.001
Marital status, n (%)			
Never married	63 (3.8)	146 (4.2)	
Divorced/ Separated	300 (18.1)	610 (17.4)	
Widowed	313 (18.9)	592 (16.9)	
Married/ Marriage-like	969 (58.5)	2,130 (60.9)	
missed	11 (0.7)	19 (0.5)	0.341
Family income ($), n (%)			
< $35,000	831 (50.2)	1,402 (40.1)	
≥ $35,000-< $50,000	318 (19.2)	687 (19.7)	
≥ $50,000-< $75,000	261 (15.8)	634 (18.1)	
≥ 75,000	194 (11.7)	658 (18.8)	
missed	52 (3.1)	115 (3.3)	< 0.001
Cancer before enrollment, n (%)	70 (4.3)	153 (4.4)	0.791
Cardiovascular disease before enrollment. n (%)	242 (16.0)	469 (14.7)	0.240
Smoking, n(%) never	845 (51.03)	1,856 (53.07)	
former	645 (38.95)	1,322 (37.80)	
current	144 (8.70)	278 (7.95)	0.468
Pack-years of cigarette consumption	11.64 (20.96)	11.64 (25.58)	0.259
Hormone therapy use ever, n (%)	667 (40.28)	1,794 (51.30)	< 0.001
Age at menopause, n (%) < 45	396 (32.49)	864 (31.10)	
45–49	441 (36.18)	985 (35.46)	
≥ 50	382 (31.34)	929 (33.44)	0.410
Alcohol intake, serves /wk	3 (2)	4 (3)	< 0.001
Coffee/tea intake cups/day	1.17 (1.59)	2.34 (1.59)	0.003
Physical activity, MET-hrs/wk	3.75 (10.50)	6.75 (15.75)	< 0.001
Depression (≥ 5 short CES-D), n (%)	289 (17.50)	535 (15.39)	0.054

Data are medians and interquartile ranges, unless otherwise specified. P values from Wilcoxon rank test for continuous variables, and Chi-square test and t-test for categorical ones

*Abbreviations*: *HDL* High density lipoprotein, *HRT* Hormone replacement therapy, *MET* Metabolic equivalent, *CES-D* Center for epidemiological studies of depression

^a^ Six women had missing information on MS at baseline

^b^ Ninety six women had missing information on insomnia at baseline

Cross-sectional analysis showed positive associations of baseline sleep duration of 8- < 9 h with WC > 88 cm (OR = 1.18; 95%CI 1.01–1.40) and TG ≥ 150 mg/dL (OR = 1.23; 95%CI 1.05–1.46), and of sleep duration ≥ 9 h with MetS (OR = 1.51; 95%CI 1.12–2.04), WC > 88 cm (OR = 1.40; 95%CI 1.04–1.90) and TG ≥ 150 mg/dL (OR = 1.42; 95% CI 1.04–1.92) (Table 2). In the fully adjusted models there was no association between sleep duration and FG ≥ 100 mg/dL, HDL < 50 mg/dL and hypertension. Using sleep duration 7- < 9 h as the referent group showed similar results (Supplementary Table 2). Insomnia had a borderline positive association with MetS (OR = 1.14; 95% CI 0.99–1.31) and a significant positive association with WC > 88 cm (OR = 1.18; 95%CI 1.03–1.34) and FG ≥ 100 mg/dL (OR = 1.17; 95%CI 1.02–1.35). When BMI was included in the model, similar results were observed for the association of sleep duration with MetS and its components, while insomnia was no longer associated with these outcomes (Supplementary Table 3).

**Table 2:** Cross-sectional analysis of association of sleep duration and insomnia with metabolic syndrome and its components using logistic regression models

	**MetS**		**Waist > 88 cm**		**Hypertension**		**Fasting glucose ≥ 100 mg/dL**		**HDL < 50 mg/dL**		**Triglycerides ≥ 150 mg/dL**	
	Model 1	Model 2	Model 1	Model 2	Model 1	Model 2	Model 1	Model 2	Model 1	Model 2	Model 1	Model 2
	OR (95%CI)	OR (95%CI)	OR (95%CI)	OR (95%CI)	OR (95%CI)	OR (95%CI)	OR (95%CI)	OR (95%CI)	OR (95%CI)	OR (95%CI)	OR (95%CI)	OR (95%CI)
**Baseline sleep duration (hours)**												
< 6	1.01 (0.83–1.24)	0.98 (0.80–1.20)	1.05 (0.87–1.28)	1.03 (0.84–1.25)	0.97 (0.80–1.18)	0.97 (0.79–1.18)	1.16 (0.94–1.43)	1.11 (0.89–1.37)	0.95 (0.77–1.18)	0.92 (0.74–1.14)	1.06 (0.86–1.29)	1.05 (0.85–1.29)
6- < 7	1.04 (0.90–1.21)	1.03 (0.89–1.21)	1.13 (0.98–1.31)	1.13 (0.98–1.31)	0.99 (0.86–1.15)	0.99 (0.85–1.15)	0.99 (0.85–1.17)	0.97 (0.82–1.15)	1.00 (0.86–1.17)	1.00 (0.85–1.17)	1.01 (0.87–1.18)	1.00 (0.86–1.16)
7- < 8	ref	ref	ref	ref	ref	ref	ref	ref	ref	ref	ref	ref
8- < 9	1.06 (0.90–1.26)	1.05 (0.89–1.25)	1.14 (0.97–1.34	1.18 (1.01–1.40)	0.91 (0.77–1.06)	0.90 (0.77–1.06)	1.01 (0.84–1.21)	0.98 (0.82–1.18)	0.94 (0.79–1.13)	0.97 (0.81–1.17)	1.22 (1.04–1.44)	1.23 (1.05–1.46)
≥ 9	1.55 (1.15–2.08)	1.51 (1.12–2.04)	1.41 (1.05–1.89)	1.40 (1.04–1.90)	1.35 (1.00–1.87)	1.34 (0.98–1.82)	1.14 (0.83–1.57)	1.08 (0.78–1.50)	1.26 (0.92–1.72)	1.28 (0.93–1.76)	1.42 (1.06–1.92)	1.42 (1.04–1.92)
**Baseline sleep quality**												
Insomnia	1.14 (1.00–1.30)	1.14 (0.99–1.31)	1.17 (1.03–1.32)	1.18 (1.03–1.34)	1.07 (0.95–1.22)	1.09 (0.96–1.24)	1.18 (1.03–1.35)	1.17 (1.02–1.35)	1.02 (0.89–1.17)	1.03 (0.89–1.18)	1.10 (0.97–1.26)	1.11 (0.97–1.26)

*Abbreviations*: *OR* Odds ratio, *CI* Confidence interval, *MetS* Metabolic syndrome, *HDL* High density lipoprotein, *ref* reference group

Model 1 adjusted for age, ethnicity (white, black, other), and study participation (observational vs. clinical trial)

Model 2 adjusted as model 1 plus educational level (less than high school grad, high school grad/some college, college grad, post-college), study participation (observational study, intervention vs. placebo arm of clinical trial), hormone therapy ever, smoking status (never, current and former), pack-years of smoking, alcohol intake (serves/wk), physical activity (MET hours/week), coffee intake (cups/day), depression (≥ 5 short CES-D), age at menopause, and marital status (currently married, never married, divorced, widowed)

In the analysis stratified by baseline insomnia status (Table 3), there were positive associations between long sleep duration (≥ 9 h) and MetS in both strata of insomnia (OR_≥9 h/insomnia_ = 2.89; 95%CI 1.39–6.01 and OR_≥9 h/no-insomnia_ = 1.54; 95%CI 1.03–2.00 respectively). There were positive associations of sleep duration ≥ 9 h with WC > 88 cm and TG ≥ 150 mg/dL among women without insomnia (OR = 1.42; 95%CI 1.02–1.98 and OR = 1.47; 95%CI 1.04–2.03 respectively) (Table 3). Short sleep duration (6- < 7 h) and insomnia were associated with WC > 88 cm (OR = 1.31; 95% CI 1.06–1.61), while the odds of having a FG ≥ 100 mg/dL was higher among those with no insomnia and < 6 h sleep duration (OR = 1.38; 95%CI 1.00–1.90) and for those with insomnia and 7- < 8 h of sleep (OR = 1.36; 95%CI 1.05–1.76).

**Table 3:** Joint cross-sectional association of sleep hours and insomnia with metabolic syndrome and its components at baseline in a sample of women from the Women’s Health Initiative using logistic regression models

	**MetS**		**Waist > 88 cm**		**Hypertension**		**Fasting glucose ≥ 100 mg/dL**		**HDL < 50 mg/dL**		**Triglycerides ≥ 150 mg/dL**	
Sleep Duration (hours)	No Insomnia	Insomnia	No Insomnia	Insomnia	No Insomnia	Insomnia	No Insomnia	Insomnia	No Insomnia	Insomnia	No Insomnia	Insomnia
	OR (95%CI)	OR (95%CI)	OR (95%CI)	OR (95%CI)	OR (95%CI)	OR (95%CI)	OR (95%CI)	OR (95%CI)	OR (95%CI)	OR (95%CI)	OR (95%CI)	OR (95%CI)
< 6	1.07 (0.79–1.47)	1.03 (0.79–1.33)	0.97 (0.71–1.32)	1.20 (0.94–1.54)	0.92 (0.68–1.24)	1.01 (0.79–1.29)	1.38 (1.00–1.90)	1.16 (0.89–1.51)	1.03 (0.74–1.42)	0.87 (0.67–1.13)	1.02 (0.74–1.41)	1.06 (0.82–1.36)
6- < 7	0.98 (0.81–1.19)	1.20 (0.98–1.49)	1.03 (0.88–1.27)	1.31 (1.06–1.61)	0.97 (0.81–1.16)	1.01 (0.82–1.25)	1.01 (0.83–1.23)	1.13 (0.90–1.43)	1.02 (0.84–1.24)	1.01 (0.80–1.24)	0.94 (0.78–1.13)	1.16 (0.92–1.43)
7- < 8	ref	1.22 (0.95–1.56)	ref	1.17 (0.92–1.48)	ref	1.08 (0.85–1.36)	ref	1.36 (1.05–1.76)	ref	1.09 (0.85–1.41)	ref	1.10 (0.87–1.40)
8- < 9	1.12 (0.92–1.35)	1.26 (0.86–1.84)	1.21 (1.01–1.57)	1.37 (0.95–1.99)	0.89 (0.74–1.06)	1.10 (0.77–1.59)	1.04 (0.85–1.28)	1.24 (0.83–1.84)	0.93 (0.76–1.14)	1.23 (0.83–1.81)	1.21 (1.01–1.46)	1.53 (1.06–2.20)
≥ 9	1.54 (1.03–2.00)	2.89 (1.39–6.01)	1.42 (1.02–1.98)	1.83 (0.87–3.86)	1.30 (0.94–1.84)	1.51 (0.71–3.24)	1.18 (0.82–1.68)	1.33 (0.61–2.91)	1.21 (0.85–1.72)	1.98 (0.79–3.57)	1.47 (1.04–2.03)	1.35 (0.54–3.41)

*Abbreviations:  MetS *Metabolic syndrome,* HDL *high density lipoproteins,* OR* Odds ratio, *CI* Confidence interval, *ref *reference group

Models adjusted for age, ethnicity, educational level, study participation, hormone therapy ever, smoking status, pack-years of smoking, alcohol intake, physical activity, coffee intake, depression, age at menopause and marital status (see Table 2 for details)

In the longitudinal analysis, there was a significant positive association between persistently short duration of sleep (< 6 h average) and the odds of developing hypertension (OR = 1.35; 95%CI 1.01–1.91) (Table 4); however, this association was no longer significant after adjustment for BMI (OR = 1.34; 95% CI 0.97–1.84) (Supplementary Table 4). In the model adjusted for age, race and WHI study arm, a reduction in sleep duration over time was associated with an increased risk of MetS (OR = 1.62; 95%CI 1.00–2.61), although this result was not significant in the fully adjusted model (OR = 1.47; 95%CI 0.90–2.37). Using 7- < 9 h of sleep as the referent category attenuated some of the associations observed in the main analysis (Supplementary Table 5).

**Table 4:** Longitudinal analysis of sleep duration and insomnia with the risk of metabolic syndrome and its components using generalized estimating equation models

	**MetS**		**Waist > 88 cm**		**Hypertension**	
	Model 1	Model 2	Model 1	Model 2	Model 1	Model 2
**Average sleep hours**	OR (95%CI)	OR (95%CI)	OR (95%CI)	OR (95%CI) ^a^	OR (95%CI)	OR (95%CI) ^a^
< 6	1.11 (0.83–1.53)	0.98 (0.70–1.37)	1.06 (0.75–1.49)	1.04 (0.74–1.48)	1.41 (1.03–1.92)	1.35 (1.01–1.91)
6- < 7	1.16 (0.90–1.49)	1.11 (0.86–1.42)	0.92 (0.70–1.20)	0.85 (0.65–1.11)	1.26 (0.99- 1.61)	1.24 (0.97–1.58)
7- < 8	ref	ref	ref	ref	ref	ref
8- < 9	1.14 (0.82–1.59)	1.13 (0.82–1.59)	0.62 (0.41–0.93)	0.62 (0.41–0.94)	1.05 (0.75–1.45)	1.02 (0.73–1.42)
≥ 9	0.62 (0.22–1.78)	0.68 (0.23–1.90)	0.40 (0.11–1.42)	0.43 (0.12–1.54)	1.00 (0.41–2.45)	1.09 (0.45–2.66)
> 2 h increase	1.27 (0.74–2.18)	1.15 (0.67–2.00)	0.83 (0.40–1.70)	0.82 (0.40–1.69)	1.22 (0.68–2.19)	1.20 (0.66–2.17)
> 2 h decrease	1.62 (1.00–2.62)	1.47 (0.90–2.37)	0.55 (0.25–1.20)	0.55 (0.25–1.21)	1.41 (0.83–2.38)	1.39 (0.82–2.37)
**Quality of sleep over time**						
Stable restful	ref	ref	ref	ref	ref	ref
Restful to insomnia	1.44 (1.04–1.99)	1.40 (1.01–1.94)	1.04 (0.70–1.53)	1.07 (0.72–1.59)	1.26 (0.91–1.74)	1.27 (0.91–1.77)
Insomnia to restful	1.16 (0.84–1.61)	1.14 (0.82–1.58)	1.34 (0.95–1.89)	1.28 (0.90–1.82)	0.81 (0.57–1.14)	0.80 (0.57–1.14)
Persistent insomnia	1.39 (1.07–1.82)	1.31 (0.99–1.73)	1.18 (0.86–1.61)	1.05 (0.75–1.47)	1.12 (0.87–1.46)	1.10 (0.84–1.46)
	**Fasting glucose ≥ 100 mg/dL**		**Triglycerides ≥ 150 mg/dL**		**HDL < 50 mg/dL**	
**Average sleep hours**	OR (95%CI)	OR (95%CI) ^a^	OR (95%CI)	OR (95%CI) ^a^	OR (95%CI)	OR (95%CI) ^a^
< 6	1.47 (0.87–2.49)	1.19 (0.90–1.59)	0.85 (0.62–1.17)	0.79 (0.57–1.09)	1.17 (0.81–1.68)	1.06 (0.73–153)
6- < 7	1.10 (0.77–1.57)	1.06 (0.85–1.32)	0.92 (0.73–1.16)	0.89 (0.70–1.13)	1.28 (0.97–169)	1.26 (0.96–1.66)
7- < 8	ref	ref	ref	ref	ref	ref
8- < 9	0.97 (0.64–1.44)	1.09 (0.81–1.47)	0.88 (0.63–1.22)	0.88 (0.63–1.22)	1.23 (0.85–1.77)	1.26 (0.87–1.82)
≥ 9	1.09 (0.42–2.85)	1.46 (0.71–3.03)	0.56 (0.21–1.51)	0.56 (0.21–1.51)	1.19 (0.46–3.04)	1.26 (0.49–3.24)
> 2 h increase	1.13 (0.62–2.06)	0.94 (0.57–1.56)	1.37 (0.84–2.23)	1.32 (0.81–2.15)	0.90 (0.44–1.85)	0.92 (0.45–1.90)
> 2 h decrease	0.92 (0.54–1.55)	0.96 (0.60–1.54)	0.98 (0.59–1.62)	0.98 (0.59–1.62)	1.37 (0.77–2.43)	1.26 (0.70–2.29)
**Quality of sleep over time**						
Stable restful	ref	ref	ref	ref	ref	ref
Restful to insomnia	1.22 (0.92–1.61)	1.24 (0.93–1.65)	1.51 (1.10–2.06)	1.48 (1.08–2.03)	1.07 (0.73–1.57)	1.00 (0.68–1.47)
Insomnia to restful	1.21 (0.92–1.58)	1.20 (0.91–1.58)	0.92 (0.67–1.26)	0.92 (0.67–1.27)	0.99 (0.68–1.43)	1.06 (0.72–1.54)
Persistent insomnia	1.21 (0.95–1.53)	1.21 (0.94–1.56)	0.96 (0.73–1.26)	0.96 (0.73–1.26)	1.28 (0.95–1.72)	1.25 (0.91–1.71)

*Abbreviations: OR* Odds ratio, *CI* Confidence interval, *MetS* metabolic syndrome, *HDL* high density lipoproteins

Model1 adjusted for age, ethnicity, and study participation

Model 2 adjusted as model 1 plus clinical trial arm, educational level, hormone therapy use, smoking status, pack-years of smoking, alcohol intake, physical activity, coffee intake, depression, age at menopause, and marital status (see Table 2 for details)

In further longitudinal analyses, change from restful sleep to insomnia was associated with increased odds of developing MetS, and TG ≥ 150 mg/dL (OR = 1.40; 95%CI 1.01–1.94 and OR = 1.48; 95%CI 1.08–2.03, respectively). Persistent insomnia was associated with increased risk of MetS, but the results were not statistically significant in the fully adjusted model (OR = 1.31; 95%CI 0.99–1.73). When BMI was included in the models, some of the associations were attenuated, while a change from insomnia to restful sleep was positively associated with WC > 88 cm (Supplementary Table 5).

## Discussion

In this study of postmenopausal women, we observed positive associations between baseline long sleep duration (≥ 9 h) and the prevalence of MetS and some of its components, including large WC and high TG levels. In addition, in the cross-sectional analysis, baseline insomnia was associated with a modest increase in the odds of WC > 88 cm and FG ≥ 100 mg/dL, and with a borderline increase in the odds of MetS. We observed greater odds of MetS in those with both longer sleep duration (≥ 9 h) and insomnia at baseline, while in those with 7- < 8 h of sleep, insomnia was associated with high FG. In longitudinal analyses, short sleep duration (< 6 h) reported on two consecutive visits, was associated with increased hypertension risk. Development of insomnia over time was associated with increased risk of MetS and high TG.

Several studies have examined the association between sleep duration and MetS, although not all of them have used the same definition of MetS [9]. The four studies that have used the NCEP/ATPIII criteria [11, 30–32] have shown inconsistent results, which may be due to relatively small sample sizes in some studies [11, 30] and limited availability of information on confounding factors in another [31]. An European study with 1,332 women found that longer sleep duration (≥ 9 h) was associated with increased prevalence of MetS [32]. A large, cross-sectional study of individuals age > 50 years old in China used a population-specific MetS definition and found a positive association between long sleep (≥ 9 h) duration and MetS, central obesity, and elevated TG [13]. Other cross-sectional studies focusing on MetS components have reported positive associations of long (≥ 8 h and ≥ 9 h in two separate studies) [33, 34] and short sleep duration (≤ 6 h) [34, 35] with TG, and of short sleep duration (< 5 h and < 6 h in two separate studies) with WC [36, 37]. Furthermore, long resting time in bed (≥ 9.5 h) appears correlated with increased lethargy, reduced physical activity, and elevation of inflammatory markers [38], while among postmenopausal women, long sleep duration (> 9 h) is associated with sarcopenia, lower muscle mass [39], and deterioration of glucose metabolism [40]. Whether these factors are causes or effects is unclear; it is likely there is a bidirectional association.

Our longitudinal analysis did not show an association between sleep duration and MetS. The analysis included only women who had a second sleep measurement during follow-up and were free of MetS at baseline and the following visit. We excluded those who developed diabetes before assessment of the outcomes, which removed women at greater risk for MetS. The only previous longitudinal study that used two measurements of sleep approximately 3 years apart found positive associations between both short sleep duration and a decrease of sleep duration of ≥ 2 h with increased odds of MetS [15]. The study sample was from northern China and was significantly younger and had lower BMI compared to the one in the present study. The results of that study might be attributable to the fact that in a younger cohort, short sleep duration was associated with other risk factors for MetS such as increased stress and work load, while in this group of postmenopausal women these situations are less common.

We observed an association between persistent short sleep duration (< 6 h) and increased risk of hypertension, although the association was slightly attenuated after adjustment for BMI at baseline. Sleep duration affects stress hormones and nervous system activities which regulate BP level [41], and lack of sleep over time has been associated with an increase in BP [42]. Our results are consistent with those of previous longitudinal studies in young adults, while studies in older women have not observed such an association [43]. Our results suggest that among postmenopausal women, BMI may partially account for the association between short sleep duration and development of hypertension, as suggested by other studies [44].

The cross-sectional analyses showed positive associations of insomnia with central obesity and FG, and a borderline positive association with MetS. A meta-analysis summarizing data from cross-sectional studies using various definitions of MetS in individuals with a wide age range and diverse health conditions concluded that insomnia was associated with MetS [14]. Several studies in middle age and elderly populations have found associations similar to ours [45]. Our results of increased odds of elevated FG are consistent with previous data suggesting that poor sleep quality, and in particular sleep fragmentation, is associated with impaired glucose homeostasis [46]. Insomnia may be a sign of sleep apnea, resulting in frequent awakening and reduced restorative sleep. Data on snoring, the major sign of obstructive sleep apnea, could help to understand the nature of the link between insomnia and metabolic changes. However, more than 50% of women in this study were unaware of whether they snored.

To the best of our knowledge, ours is the first study that has examined the association of change in insomnia over time with risk of MetS. Two previous longitudinal studies tested the insomnia/sleep fragmentation association with MetS; both found positive associations, although they were conducted in smaller study populations and used only a single assessment of the exposure [47, 48]. Our results suggest the importance of evaluating changes in sleep patterns over time.

The present study was conducted in a population sampled from the WHI with detailed demographic, lifestyle, and medical information. Quality control measures were incorporated into data collection, and laboratory assays. Sleep duration and insomnia data from two distinct exams, a few years apart, allowed us to evaluate the association of change in these measures with various outcomes. The analyses were adjusted for potential confounding factors, including depression, which has been repeatedly associated with both sleep duration and insomnia [6], and MetS [49], especially among those aged ≥ 60 years, but was not accounted for in most of the previous studies.

The study was conducted on approximately 3.2% of the WHI cohort and the majority of the study population (> 80%) were participants in the WHI clinical trials. This group may not be representative of postmenopausal women in the general population, and caution should be adopted in extrapolating the results to other segments of the population. Longitudinal data were available only for a portion of the study subjects limiting the statistical power for this analysis. In this analytical sample, sleep duration and frequency of insomnia at baseline appeared similar between women with at least one follow-up exam and those with none; however, a previous study conducted in 158,203 postmenopausal women (97.8% of the WHI cohort) showed an association between both short (≤ 5 h) and long (≥ 9 h) sleep duration and mortality [6]. Participants who developed MetS over time were more likely to die or to not return for the next exam. If a selective loss to follow-up for those with short and long duration of sleep and those who developed MetS occurred, it might have caused an underestimation of the association. Sleep duration and quality were self-reported and may have been misclassified. However, most previous population-based studies have relied on self-report sleep data. More objective measures such as actigraphy and polysomnography may yield different results, although their cost prevents their application in large-scale studies.

## Conclusion

This is the first study to present cross-sectional and longitudinal analyses of both self-reported sleep duration and insomnia in association with MetS and its components. The results indicate that, among postmenopausal women, alterations of these sleep measures are associated with increased current and future risk of MetS and several of its components, highlighting the importance of maintaining healthy sleep habits.

## Supplementary Information

**Additional file 1: Supplementary Table 1.** Frequency of sleep duration and insomnia and metabolic syndrome at baseline and follow-up exams. **Supplementary table 2.** Cross-sectional analysis of sleep duration with metabolic syndrome and its components using logistic regression models and sleep duration 7-<9 hours as referent group. **Supplementary table 3.** Cross-sectional analysis of sleep duration and insomnia association with metabolic syndrome and its components adjusted for multiple confounding variables including body mass index using logistic regression models. **Supplementary table 4.** Longitudinal analysis of sleep duration and insomnia association with metabolic syndrome and its components adjusted for multiple confounding variables including body mass index using Generalized Estimating Equation models. **Supplementary table 5.** Longitudinal analysis of sleep duration with metabolic syndrome and its components using Generalized Equation models and sleep duration 7-<9 hours as referent group. **Supplementary Table 6.** List of the Women Health’s Initiative study participating centers that approved the study.

## Data Availability

Data used for the present study are the property of the National Institutes of Health. Copies of the de-identified data used in our study will be made available upon request to, and pending approval by, the Women’s Health Initiative Publications and Presentations Committee (email: p&p@WHI.org.)

## Abbreviations

MetS: Metabolic syndrome
WHI: Women’s Health Initiative
CI: Confidence interval
OR: Odds ratio
TG: Triglycerides
OS: Observational study
CT: Clinical trial
BP: Blood pressure
WC: Waist circumference
WHIIRS: WHI Insomnia Rating Score
CES-D: Center for Epidemiological Studies of Depression scale
BMI: Body mass index
NCEP/ATP-III: National Cholesterol Education Program’s Adult Treatment Panel-III report
FG: Fasting glucose
HDL: High density cholesterol
GEE: Generalized estimating equation
