# Supplementary material for: Association of sleep duration and insomnia with metabolic syndrome and its components in the Women’s Health Initiative
Source: BMC Endocr Disord. 2022 Sep 14;22:228. doi: 10.1186/s12902-022-01138-9 (PMC9476543; doi:10.1186/s12902-022-01138-9)
Supplement: Supplementary file 1 — Additional file 1: Supplementary Table 1. Frequency of sleep duration and insomnia and metabolic syndrome at baseline and follow-up exams. Supplementary table 2. Cross-sectional analysis of sleep duration with metabolic syndrome and its components using logistic regression models and sleep duration 7-<9 hours as referent group. Supplementary table 3. Cross-sectional analysis of sleep duration and insomnia association with metabolic syndrome and its components adjusted for multiple confounding variables including body mass index using logistic regression models. Supplementary table 4. Longitudinal analysis of sleep duration and insomnia association with metabolic syndrome and its components adjusted for multiple confounding variables including body mass index using Generalized Estimating Equation models. Supplementary table 5. Longitudinal analysis of sleep duration with metabolic syndrome and its components using Generalized Equation models and sleep duration 7-<9 hours as referent group. Supplementary Table 6. List of the Women Health’s Initiative study participating centers that approved the study. [file 12902_2022_1138_MOESM1_ESM.docx]

Association of sleep duration and insomnia with metabolic syndrome and its components in the Women’s Health Initiative

Supplementary Material

Supplementary Table 1. Frequency of sleep duration and insomnia and metabolic syndrome at baseline and follow-up exams

| **Exposure** | **Baseline** | **Year 1^ab^** | **Year 3^a^** | **Year 6^a^** | **Year 9^a^** |
| --- | --- | --- | --- | --- | --- |
| Sleep duration total sample | 5,159 | 2,532 | 2,830 | 3,490 | 715 |
| <6 | 621 | 301 | 336 | 441 | 64 |
| 6-<7 | 1,542 | 796 | 844 | 1,022 | 195 |
| 7-<8 | 1,781 | 895 | 1,009 | 1,164 | 249 |
| 8-<9 | 1,003 | 449 | 527 | 692 | 174 |
| ≥ 9 | 212 | 91 | 114 | 171 | 33 |
| Insomnia /total | 1,514/5,057 | 665/2,479 | 820/2,782 | 1,050/3,429 | 202/703 |
| **Outcome** |  |  |  |  |  |
| Metabolic Syndrome, yes/ total | 1,656/5,153 | 272/2460 | 180/1,970 | 145/1,468 | - |
| Waist ≥ 90 cm/ total | 2,138/5,138 | 175/2,092 | 240/2,170 | 115/1,003 | 19/121 |
| Hypertension or med./ total | 2,843/5,159 | 1,486/2,142 | 321/1,782 | 178/1,125 | 33/242 |
| Fast glucose ≥ 100 mg/dL or med./ total | 1,390/5,033 | 306/2,523 | 240/2,170 | 249/1,499 | - |
| Triglycerides ≥ 150 mg/dL/ total | 1,880/5,044 | 455/2,337 | 231/1,691 | 135/1,379 | - |
| HDL < 50 mg/dL/ total | 1,451/5,029 | 203/2,501 | 168/2,028 | 100/1,534 | - |

**^a^** Among those who were outcome free at the previous exams.

^b^ Year 1 data were available for participants to the clinical trial arm only.

Supplementary table 2. Cross-sectional analysis of sleep duration with metabolic syndrome and its components using logistic regression models and sleep duration 7-<9 hours as referent group.

|  | **MetS** | | **Waist > 88 cm** | | **Hypertension** | | **Fasting glucose ≥ 100 mg/dL** | | **HDL < 50 mg/dL** | | **Triglycerides ≥ 150 mg/dL** | |
| --- | --- | --- | --- | --- | --- | --- | --- | --- | --- | --- | --- | --- |
|  | Model 1 | Model 2 | Model 1 | Model 2 | Model 1 | Model 2 | Model 1 | Model 2 | Model 1 | Model 2 | Model 1 | Model 2 |
|  | OR (95%CI) | OR (95%CI) | OR (95%CI) | OR (95%CI) | OR (95%CI) | OR (95%CI) | OR (95%CI) | OR (95%CI) | OR (95%CI) | OR (95%CI) | OR (95%CI) | OR (95%CI) |
| **Baseline sleep duration (hours)** | | | | | | | | | | | | |
| <6 | 1.02  (0.85-1.24) | 0.94  (0.77-2.25) | 1.05  (0.87-1.26) | 0.95  (0.79-1.16) | 1.00  (0.83-1.21) | 0.97  (0.80-1.17) | 1.16  (0.95-1.41) | 1.15  (0.94-1.42) | 0.97  (0.79-1.19) | 0.93  (0.75-1.14) | 0.98  (0.81-1.20) | 0.93  (0.75-1.14) |
| 6-<7 | 1.01  (0.88-1.16) | 0.98  (0.85-1.13) | 1.05  (0.92-1.20) | 1.00  (0.88-1.15) | 1.03  (0.90-1.17) | 1.01  (0.88-1.15) | 0.99  (0.86-1.15) | 0.99  (0.85-1.15) | 1.02  (0.88-1.18) | 0.99  (0.86-1.15) | 0.94  (0.82-1.08) | 0.99  (0.86-1.15) |
| 7-<9 | ref. | ref. | ref. | ref. | ref. | ref. | ref. | ref. | ref. | ref. | ref. | ref. |
| ≥ 9 | 1.50  (1.13-2.00) | 1.46  (1.09-1.96) | 1.34  (1.00-1.78) | 1.31  (0.97-1.76) | 1.40  (1.04-1.89) | 1.35  (1.00-1.83) | 1.13  (0.82-1.55) | 1.07  (0.78-1.48) | 1.29  (0.95-1.75) | 1.25  (0.91-1.71) | 1.32  (0.99-1.78) | 1.25  (0.91-1.71) |

OR, odds ratio, CI, confidence interval.

Model 1 adjusted for age, ethnicity and study component participation.

Model 2 adjusted for age, ethnicity, study component participation, educational level, study participation, hormone therapy ever, smoking status, pack-years of smoking, alcohol intake, physical activity, coffee intake, depression, age at menopause, and marital status,

Supplementary table 3. Cross-sectional analysis of sleep duration and insomnia association with metabolic syndrome and its components adjusted for multiple confounding variables including body mass index using logistic regression models

|  | **MetS** | **Waist > 88 cm** | **Hypertension** | **Fasting glucose ≥ 100 mg/dL** | **HDL < 50 mg/dL** | **Triglycerides ≥ 150 mg/dL** |
| --- | --- | --- | --- | --- | --- | --- |
|  | OR (95%CI) | OR (95%CI) | OR (95%CI) | OR (95%CI) | OR (95%CI) | OR (95%CI) |
| **Sleep Duration (hours)**^c^ | | | | | | |
| <6 | 0.96 (0.77-1.20) | 0.99 (0.75-1.30) | 0.92 (0.75-1.13) | 1.14 (0.92-1.42) | 0.89 (0.71-1.12) | 0.99 (0.80-1.22) |
| 6-<7 | 1.01 (0.86-1.19) | 1.09 (0.89-1.34) | 0.96 (0.83-1.12) | 0.97 (0.82-1.15) | 0.99 (0.84-1.16) | 0.99 (0.85-1.15) |
| 7-<8 | ref. | ref. | ref. | ref. | ref. | ref. |
| 8-<9 | 1.08 (0.89-1.30) | 1.27 (1.00-1.59) | 0.89 (0.75-1.05) | 0.97 (0.80-1.17) | 0.97 (0.80-1.16) | 1.23 (1.04-1.45) |
| ≥ 9 | 1.45 (1.04-2.01) | 1.31 (0.87-1.96) | 1.28 (0.93-1.75) | 1.02 (0.73-1.43) | 1.18 (0.85-1.64) | 1.36 (1.00-1.85) |
| **Insomnia** ^b^ | 1.07 (0.92-1.24) | 1.12 (0.93-1.35) | 1.06 (0.92-1.21) | 1.14 (0.98-1.32) | 0.99 (0.86-11.5) | 1.05 (0.92-1.21) |

OR, odds ratio, CI, confidence interval.

Model adjusted for age, ethnicity, study component participation, educational level, study component participation, hormone therapy ever, smoking status, pack-years of smoking, alcohol intake, physical activity, coffee intake, depression, age at menopause, marital status, and body mass index (<20, 20-25, >25-<30, ≥30 kg/m^2^), (see Table 2 for details).

^a^ Model adjusted for insomnia. ^b^ Model adjusted for sleep duration.

Supplementary table 4. Longitudinal analysis of sleep duration and insomnia association with metabolic syndrome and its components adjusted for multiple confounding variables including body mass index using Generalized Estimating Equation models

|  | **MetS** | **Waist > 88 cm** | **Hypertension** | **Fasting glucose ≥ 100 mg/dL** | **Triglycerides ≥ 150 mg/dL** | **HDL < 50 mg/dL** |
| --- | --- | --- | --- | --- | --- | --- |
|  | Model 1 | Model 2 | Model 1 | Model 2 | Model 1 | Model 2 |
| **Average sleep hours** | OR (95%CI) | OR (95%CI) | OR (95%CI) | OR (95%CI) | OR (95%CI) | OR (95%CI) |
| <6 | 1.00 (0.71-1.40) | 0.99 (0.68-1.44) | 1.34 (0.97-1.84) | 1.20 (0.90-1.60) | 0.80 (0.58-1.10) | 1.00 (0.71-1.42) |
| 6-<7 | 1.14 (0.88-1.47) | 1.02 (0.76-1.35) | 1.23 (0.96-1.57) | 1.10 (0.88-1.38) | 0.91 (0.72-1.15) | 1.13 (0.87-1.45) |
| 7-<8 | ref. | ref. | ref. | ref. | ref. | ref. |
| 8-<9 | 1.15 (0.82-1.62) | 0.66 (0.42-1.02) | 1.01 (0.72-1.40) | 1.10 (0.82-1.47) | 0.91 (0.65-1.26) | 1.22 (0.87-1.71) |
| ≥9 | 0.73 (0.25.2.11) | 0.39 (0.11-1.45) | 1.10 (0.45-2.68) | 1.57 (0.75-3.29) | 0.57 (0.21-1.52) | 1.16 (0.50-2.70) |
| > 2 hrs increase | 1.02 (0.58-1.77) | 0.74 (0.35-1.55) | 1.20 (0.67-2.17) | 0.85 (0.51-1.41) | 1.31 (0.80-2.14) | 0.54 (0.24-1.23) |
| > 2 hrs decrease | 1.36 (0.82-2.24) | 0.53 (0.22-1.23) | 1.38 (0.81-2.34) | 0.94 (0.59-1.50) | 0.95 (0.57-1.57) | 1.49 (0.91-2.44) |
| Stable restful | ref. | ref. | ref. | ref. | ref. | ref. |
| Restful to insomnia | 1.41 (1.01-1.97) | 0.88 (0.58-1.35) | 1.27 (0.91-.76) | 1.20 (0.90-1.60) | 1.47 (1.07-2.02) | 1.10 (0.77-1.56) |
| Insomnia to restful | 1.15 (0.82-1.60) | 1.47 (1.01-2.12) | 0.80 (0..14) | 1.19 (0.90-1.57) | 0.91 (0.66-1.26) | 1.08 (0.76-1.54) |
| Persistent insomnia | 1.28 (0.96-1.71) | 1.19 (0.83-1.69) | 1.05 (0.80-1.39) | 1.18 (0.92-1.53) | 0.95 (0.72-1.26) | 1.20 (0.89-1.61) |

OD, odds ratio, CI, confidence interval.

Model adjusted for age, ethnicity, study component participation, educational level, study participation, hormone therapy ever, smoking status, pack-years of smoking, alcohol intake, physical activity, coffee intake, depression, age at menopause, marital status, and body mass index (<20, 20-25, >25-<30, ≥30 kg/m^2^) (see Table 2 details).

Supplementary table 5. Longitudinal analysis of sleep duration with metabolic syndrome and its components using Generalized Estimating Equation models and sleep duration 7-<9 hours as referent group

|  | Model 1 | Model 2 | Model 1 | Model 2 | Model 1 | Model 2 |
| --- | --- | --- | --- | --- | --- | --- |
|  | **MetS** | | **Waist > 88 cm** | | **Hypertension** | |
| **Average sleep hours** | OR (95%CI) | OR (95%CI) | OR (95%CI) | OR (95%CI) ^a^ | OR (95%CI) | OR (95%CI) ^a^ |
| <6 | 0.93 (0.59-1.46) | 0.81 (0.51-1.28) | 0.85 (0.51-1.42) | 0.80 (0.47-1.35) | 1.37 (0.93-2.03) | 1.41 (0.90-2.03) |
| 6-<7 | 1.13 (0.91-1.40) | 1.09 (0.87-1.37) | 1.11 (0.87-1.41) | 1.07 (0.84-1.37) | 1.28 (1.03-1.58) | 1.27 (1.02-1.59) |
| 7-<9 | ref. | ref. | ref. | ref. | ref. | ref. |
| ≥9 | 0.60 (0.21-1.70) | 0.70 (0.24-2.03) | 0.45 (0.13-1.60) | 0.47 (0.13-1.71) | 0.99 (0.41-2.41) | 1.09 (0.45-2.65) |
| > 2 hrs increase | 1.21 (0.71-2.06) | 0.98 (0.57-1.700 | 0.93 (0.45-1.90) | 0.91 (0.44-1.88) | 1.20 (0.67-2.14) | 1.24 (0.69-2.23) |
| > 2 hrs decrease | 1.56 (0.97-2.50) | 1.28 (0.79-2.09) | 0.62 (0.29-1.35) | 0.61 (0.28-1.35) | 1.38 (0.82-2.32) | 1.38 (0.81-2.30) |
|  |  |  |  |  |  |  |
|  | **Fasting glucose ≥ 100 mg/dL** | | **Triglycerides ≥ 150 mg/dL** | | **HDL < 50 mg/dL** | |
| **Average sleep hours** | OR (95%CI) | OR (95%CI) ^a^ | OR (95%CI) | OR (95%CI) ^a^ | OR (95%CI) | OR (95%CI) ^a^ |
| <6 | 1.17 (0.62-2.22) | 1.09 (0.74-1.61) | 0.94 (0.63-1.41) | 0.88 (0.58-1.34) | 1.00 (0.61-1.66) | 0.93 (0.56-1.54) |
| 6-<7 | 1.10 (0.77-1.57) | 1.09 (0.90-1.33) | 0.93 (0.75-1.14) | 0.90 (0.73-1.12) | 1.13 (0.88-1.45) | 1.12 (0.87-1.44) |
| 7-<9 | ref. | ref. | ref. | ref. | ref. | ref. |
| ≥9 | 1.13 (0.46-2.79) | 1.52 (0.73-3.18) | 0.53 (0.20-1.41) | 0.58 (0.22-1.55) | 0.97 (0.37-2.56) | 1.04 (0.40-2.72) |
| > 2 hrs increase | 1.02 (0.56-1.84) | 0.82 (0.49-1.35) | 1.46 (0.90-2.35) | 1.34 (0.83-2.19) | 0.77 (0.36-1.62) | 0.76 (0.36-1.60) |
| > 2 hrs decrease | 0.94 (0.58-1.51) | 0.91 (0.57-1.44) | 1.13 (0.69-1.85) | 0.98 (0.59-1.61) | 1.26 (0.70-2.27) | 1.15 (0.64-2.07) |

OD, odds ratio, CI, confidence interval.

Model 1 adjusted for age, ethnicity, and study component participation.

Model 2 adjusted for age, ethnicity, study component participation, educational level, study participation, hormone therapy ever, smoking status, pack-years of smoking, alcohol intake, physical activity, coffee intake, depression, age at menopause, and marital status (see Table 2 details).

Supplementary Table 6

List of the Women Health’s Initiative study participating centers that approved the study

| **Study center** | **Location** |
| --- | --- |
| Emory University Atlanta | Decatur, Georgia |
| University of Alabama at Birmingham | Birmingham, Alabama |
| Bowman Gray School of Medicine Winston-Salem | Greensboro, North Carolina |
| *Brigham and Women’s Hospital* | *Boston (Chestnut Hill), Massachusetts* |
| State University of New York, Buffalo | Buffalo, New York |
| Northwestern University | Chicago and Evanston, Illinois |
| University of Iowa | Iowa City and Bettendorf, Iowa |
| University of California, San Diego | La Jolla and Chula Vista, California |
| University of Tennessee | Memphis, Tennessee |
| University of Minnesota | Minneapolis, Minnesota |
| University of Medicine and Dentistry | Newark, New Jersey |
| Memorial Hospital of Rhode Island | Pawtucket, Rhode Island |
| University of Pittsburgh | Pittsburgh, Pennsylvania |
| Fred Hutchinson Cancer Research Center | Seattle, Washington |
| University of Arizona | Tucson and Phoenix, Arizona |
| University of California | Davis, Sacramento, California |
| University of North Carolina at Chapel Hill | Chapel Hill, North Carolina |
| Rush Presbyterian- St. Luke’s Medical Center | Chicago, Illinois |
| University of Cincinnati | Cincinnati, Ohio |
| Ohio State University | Columbus, Ohio |
| Wayne State University | Detroit, Michigan |
| University of Florida | Gainesville and Jacksonville, Florida |
| George Washington University | Washington, DC |
| University of Hawaii | Honolulu, Hawaii |
| Baylor College of Medicine | Houston, Texas |
| University of California, Irvine | Irvine, California |
| University of California, Los Angeles | Los Angeles, California |
| University of Wisconsin | Madison, Wisconsin |
| Medlantic Research Institute | Washington, DC |
| University of Miami | Miami, Florida |
| Medical College of Wisconsin | Milwaukee, Wisconsin |
| University of Nevada | Reno, Nevada |
| Albert Einstein College of Medicine | Bronx, New York |
| Kaiser Foundation Research Institute | Oakland, California |
| Kaiser Foundation Research Institute | Portland, Oregon |
| University of Texas | San Antonio, Texas |
| Stanford University | San Jose, California |
| Research Foundation of SUNY, Stony Brook | Stony Brook, New York |
| University of California, Los Angeles | Torrance, California |
| University of Massachusetts | Worcester, Massachusetts |

**Acknowledgments**

List of the main [Women's Health](https://www.sciencedirect.com/topics/medicine-and-dentistry/womens-health) Initiative Investigators.

**Program Office**: (National Heart, Lung, and Blood Institute, Bethesda, MD) Jacques Roscoe, Shari Ludlum, Dale Burden, Joan McGowan, Leslie Ford, and Nancy Geller.

**Clinical Coordinating Center:** (Fred Hutchinson Cancer Research Center, Seattle, WA) Garnet Anderson, Ross Prentice, Andrea LaCroix, and Charles Kopperberg).

**Investigators and Academic Centers**: (Brigham and Women's Hospital, Harvard Medical School, Boston, MA) JoAnn E, Manson; (MedStar Health Research Institute/Howard University, Washington, DC) Barbara V Howard; (Stanford Prevention Research Center, Stanford, CA) Marcia L. Stefanick; (The Ohio State University, Columbus, OH) Rebecca Jackson; (University of Arizona, Tucson/Phoenix, AZ) Cynthia A. Thompson; (University at Buffalo, Buffalo, NY) Jean Wactawski-Wende; (University of Florida, Gainesville/Jacksonville, FL) Marian Limacher; (University of Iowa, Iowa City/Davenport, IA) Robert Wallace; (University of Pittsburgh, Pittsburgh, PA) Lewis Kuller; (City of Hope Comprehensive Cancer Center, Duarte, CA) Rowan T. Chlebowski; (Wake Forest University School of Medici ne, Winston–Salem, NC) Sally Shumaker.

[Women's Health](https://www.sciencedirect.com/topics/medicine-and-dentistry/womens-health) Initiative Memory Study: (Wake Forest University School of Medicine, Winston Salem, NC) Sally Shumaker.

**Additional information**: A full list of all the investigators who have contributed to [Women's Health](https://www.sciencedirect.com/topics/medicine-and-dentistry/womens-health) Initiative science appears at: <https://www.whi.org/researchers/Documents%20%20Write%20a%20Paper/WHI%20Investigator%20Long%20List.pdf>.
